# Diiodidobis(1-methyl­imidazole-κ*N* ^3^)cadmium(II)

**DOI:** 10.1107/S1600536808030225

**Published:** 2008-09-24

**Authors:** Juan Zhao

**Affiliations:** a College of Mechanical Engineering, Qingdao Technological University, Qingdao 266033, People’s Republic of China

## Abstract

In the title compound, [CdI_2_(C_4_H_6_N_2_)_2_], each Cd atom is coordinated by two N atoms from two 1-methylimidazole and two iodido ligands. The Cd atom has a distorted tetrahedral coordination. Inter­molecular C—H⋯I hydrogen bonds link the monomeric units, generating a one-dimensional supra­molecular chain along the *a* axis.

## Related literature

For a related structure, see: Chand *et al.* (2003 ▶).
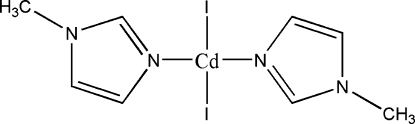


## Experimental

### 

#### Crystal data

[CdI_2_(C_4_H_6_N_2_)_2_]
*M* *_r_* = 530.43
Orthorhombic, 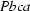

*a* = 13.5570 (9) Å
*b* = 14.5615 (14) Å
*c* = 14.9585 (19) Å
*V* = 2953.0 (5) Å^3^
*Z* = 8
Mo *K*α radiation
μ = 5.64 mm^−1^
*T* = 298 K
0.10 × 0.10 × 0.10 mm

#### Data collection

Bruker SMART 1K CCD area-detector diffractometer
Absorption correction: multi-scan (*SADABS*; Sheldrick, 2004 ▶) *T*_min_ = 0.574, *T*_max_ = 0.579
2888 measured reflections
2768 independent reflections
1811 reflections with *I* > 2σ(*I*)
*R*_int_ = 0.013

#### Refinement

*R*[*F* ^2^ > 2σ(*F* ^2^)] = 0.063
*wR*(*F* ^2^) = 0.178
*S* = 0.98
2768 reflections
137 parameters
40 restraints
H-atom parameters constrained
Δρ_max_ = 1.18 e Å^−3^
Δρ_min_ = −0.85 e Å^−3^

### 

Data collection: *SMART* (Bruker, 2001 ▶); cell refinement: *SAINT* (Bruker, 2001 ▶); data reduction: *SAINT*; program(s) used to solve structure: *SHELXTL* (Sheldrick, 2008 ▶); program(s) used to refine structure: *SHELXTL*; molecular graphics: *SHELXTL*; software used to prepare material for publication: *SHELXTL* and local programs.

## Supplementary Material

Crystal structure: contains datablocks global, I. DOI: 10.1107/S1600536808030225/bq2097sup1.cif

Structure factors: contains datablocks I. DOI: 10.1107/S1600536808030225/bq2097Isup2.hkl

Additional supplementary materials:  crystallographic information; 3D view; checkCIF report

## Figures and Tables

**Table 1:** Hydrogen-bond geometry (Å, °)

*D*—H⋯*A*	*D*—H	H⋯*A*	*D*⋯*A*	*D*—H⋯*A*
C5—H5*B*⋯I1^i^	0.96	3.03	3.9797	169

Symmetry code: (i) 
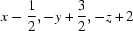
.

**Fig. 1.**
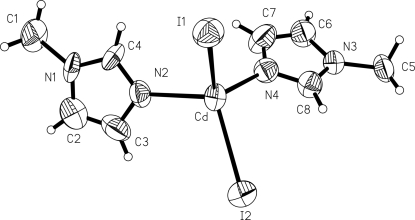
The molecular structure of (I), showing 50% probability displacement ellipsoids and the atom-numbering scheme.

**Fig. 2.**
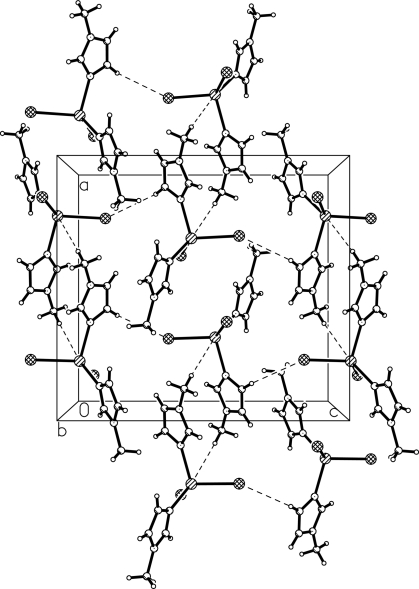
The packing of (I), viewed down the b-axis.

## supplementary crystallographic information

### Comment

In the title compound (I) (Fig. 1), each Cd atom is tetrahedrally surrounded showing a CdN_2_Cl_2_ coordination sphere. Each Mim(Mim = *N*-methylimidazole) acts as a monodentate-*N*(imidazole) donor ligand. The two imidazole rings are planar and make a dihedral angle of 69.46 (3)°. The Cd—*N*(imidazole) distance [Cd—N2, 2.238 (9); Cd—N4, 2.201 (10)°] is comparable with reported data (Chand, *et al.*, 2003). The Cd—I bond distances are 2.7248 (13)Å and 2.7358 (13)Å. The angles extended in tetrahedral CdN_2_I_2_ geometry are I1—Cd—I2 119.20 (5)°, N4—Cd—N2, 112.2 (4)° and suggest a small distortion. All other angles are within the limits of distorted Td-geometry. Intermolecular C—H···I hydrogen bonds link the monomeric units to produce a one-dimensional supramolecular chain along the *a*-axis.

In the corresponding copper compound [Cd(HaaiMe)_2_Cl_2_] (Chand, *et al.*, 2003), the Cd^II^ has a distorted tetrahedron coordination environment.

### Experimental

*N*-Methylimidazole (32.8 mg, 0.4 mmol) in MeOH (10 ml) was added in dropwise to a stirred methanolic solution (10 ml) of CdI_2_ (366.2 mg, 0.1 mmol) at room temperature (298 K). The colorless solution was left undisturbed for 2 weeks. Colorless crystals were obtained. These were then washed with water and finally, dried *in vacuo*.

### Refinement

H atoms were positioned geometrically (C—H = 0.93Å or 0.96 Å) and allowed to ride on their parent atoms with *U*_iso_(H) = 1.2 or 1.5 times *U*_eq_(C).

### Crystal data

**Table:**

[CdI_2_(C_4_H_6_N_2_)_2_]	*F*(000) = 1936
*M**_r_* = 530.43	*D*_x_ = 2.386 Mg m^−^^3^
Orthorhombic, *P**b**c**a*	Mo *K*α radiation, λ = 0.71073 Å
Hall symbol: -P 2ac 2ab	Cell parameters from 25 reflections
*a* = 13.5570 (9) Å	θ = 10–14°
*b* = 14.5615 (14) Å	µ = 5.64 mm^−^^1^
*c* = 14.9585 (19) Å	*T* = 298 K
*V* = 2953.0 (5) Å^3^	Block, colorless
*Z* = 8	0.10 × 0.10 × 0.10 mm

### Data collection

**Table:**

Bruker SMART 1K CCD area-detector diffractometer	2768 independent reflections
Radiation source: fine-focus sealed tube	1811 reflections with *I* > 2σ(*I*)
graphite	*R*_int_ = 0.013
Thin–slice ω scans	θ_max_ = 26.0°, θ_min_ = 2.5°
Absorption correction: multi-scan (SADABS; Sheldrick, 2004)	*h* = 0→16
*T*_min_ = 0.574, *T*_max_ = 0.579	*k* = 0→17
2888 measured reflections	*l* = 0→18

### Refinement

**Table:**

Refinement on *F*^2^	Secondary atom site location: difference Fourier map
Least-squares matrix: full	Hydrogen site location: inferred from neighbouring sites
*R*[*F*^2^ > 2σ(*F*^2^)] = 0.063	H-atom parameters constrained
*wR*(*F*^2^) = 0.178	*w* = 1/[σ^2^(*F*_o_^2^) + (0.1*P*)^2^ + 1*P*] where *P* = (*F*_o_^2^ + 2*F*_c_^2^)/3
*S* = 0.99	(Δ/σ)_max_ = 0.001
2768 reflections	Δρ_max_ = 1.18 e Å^−^^3^
137 parameters	Δρ_min_ = −0.85 e Å^−^^3^
40 restraints	Extinction correction: SHELXTL (Sheldrick, 2001), Fc^*^=kFc[1+0.001xFc^2^λ^3^/sin(2θ)]^-1/4^
Primary atom site location: structure-invariant direct methods	Extinction coefficient: 0.0017 (2)

### Special details

**Table:**

Geometry. All e.s.d.'s (except the e.s.d. in the dihedral angle between two l.s. planes) are estimated using the full covariance matrix. The cell e.s.d.'s are taken into account individually in the estimation of e.s.d.'s in distances, angles and torsion angles; correlations between e.s.d.'s in cell parameters are only used when they are defined by crystal symmetry. An approximate (isotropic) treatment of cell e.s.d.'s is used for estimating e.s.d.'s involving l.s. planes.
Refinement. Refinement of *F*^2^ against ALL reflections. The weighted *R*-factor *wR* and goodness of fit *S* are based on *F*^2^, conventional *R*-factors *R* are based on *F*, with *F* set to zero for negative *F*^2^. The threshold expression of *F*^2^ > σ(*F*^2^) is used only for calculating *R*-factors(gt) *etc*. and is not relevant to the choice of reflections for refinement. *R*-factors based on *F*^2^ are statistically about twice as large as those based on *F*, and *R*- factors based on ALL data will be even larger.

### Fractional atomic coordinates and isotropic or equivalent isotropic displacement parameters (Å^2^)

**Table:**

	*x*	*y*	*z*	*U*_iso_*/*U*_eq_	
Cd	1.20251 (6)	0.53829 (6)	1.04405 (6)	0.0520 (3)	
I1	1.21055 (7)	0.59157 (7)	0.86882 (6)	0.0621 (3)	
C1	1.5970 (10)	0.6525 (10)	1.0696 (12)	0.080 (5)	
H1A	1.5798	0.7004	1.0284	0.120*	
H1B	1.6475	0.6146	1.0439	0.120*	
H1C	1.6208	0.6792	1.1242	0.120*	
N1	1.5119 (6)	0.5977 (7)	1.0881 (7)	0.052 (3)	
I2	1.13534 (7)	0.36699 (7)	1.08491 (8)	0.0707 (4)	
N2	1.3612 (7)	0.5502 (7)	1.0836 (7)	0.052 (3)	
C2	1.5085 (12)	0.5226 (11)	1.1438 (10)	0.070 (4)	
H2A	1.5602	0.4954	1.1750	0.084*	
N3	0.9637 (7)	0.6773 (6)	1.1774 (6)	0.047 (2)	
C3	1.4143 (12)	0.4979 (10)	1.1428 (10)	0.072 (4)	
H3A	1.3877	0.4512	1.1778	0.087*	
N4	1.1068 (8)	0.6296 (7)	1.1229 (7)	0.052 (2)	
C4	1.4208 (9)	0.6106 (9)	1.0530 (9)	0.054 (3)	
H4A	1.4040	0.6566	1.0126	0.065*	
C5	0.8536 (8)	0.6817 (10)	1.2037 (9)	0.061 (4)	
H5A	0.8204	0.6280	1.1819	0.092*	
H5B	0.8242	0.7355	1.1779	0.092*	
H5C	0.8479	0.6844	1.2676	0.092*	
C6	1.0338 (10)	0.7448 (10)	1.1951 (9)	0.066 (3)	
H6A	1.0230	0.8005	1.2238	0.080*	
C7	1.1210 (11)	0.7135 (11)	1.1622 (11)	0.078 (4)	
H7A	1.1810	0.7443	1.1658	0.093*	
C8	1.0131 (9)	0.6115 (9)	1.1374 (8)	0.054 (3)	
H8A	0.9841	0.5563	1.1206	0.064*	

### Atomic displacement parameters (Å^2^)

**Table:**

	*U*^11^	*U*^22^	*U*^33^	*U*^12^	*U*^13^	*U*^23^
Cd	0.0379 (5)	0.0590 (5)	0.0591 (6)	0.0005 (4)	0.0060 (4)	0.0008 (5)
I1	0.0644 (6)	0.0656 (6)	0.0564 (5)	−0.0062 (4)	−0.0039 (4)	0.0073 (4)
C1	0.046 (8)	0.069 (9)	0.125 (14)	−0.023 (7)	−0.017 (8)	−0.024 (9)
N1	0.024 (5)	0.064 (6)	0.068 (7)	−0.011 (4)	0.009 (5)	−0.022 (6)
I2	0.0632 (6)	0.0605 (6)	0.0883 (8)	−0.0060 (4)	0.0029 (5)	0.0166 (5)
N2	0.037 (5)	0.052 (6)	0.067 (7)	0.003 (5)	−0.008 (5)	−0.009 (5)
C2	0.068 (10)	0.081 (11)	0.062 (9)	0.013 (8)	−0.010 (8)	−0.012 (8)
N3	0.041 (4)	0.050 (5)	0.049 (5)	0.002 (4)	0.001 (4)	−0.001 (4)
C3	0.081 (11)	0.064 (8)	0.073 (10)	0.027 (8)	0.003 (8)	0.016 (8)
N4	0.052 (5)	0.056 (5)	0.050 (5)	0.000 (4)	0.003 (4)	0.002 (4)
C4	0.038 (6)	0.065 (8)	0.059 (8)	−0.022 (6)	0.010 (6)	0.006 (6)
C5	0.040 (7)	0.076 (9)	0.068 (9)	0.014 (6)	−0.001 (6)	0.009 (7)
C6	0.060 (6)	0.056 (5)	0.083 (8)	−0.001 (5)	−0.007 (6)	−0.013 (5)
C7	0.069 (6)	0.071 (6)	0.094 (8)	−0.022 (5)	0.015 (6)	−0.009 (6)
C8	0.052 (5)	0.055 (5)	0.054 (6)	0.002 (4)	0.007 (5)	−0.010 (5)

### Geometric parameters (Å, °)

**Table:**

Cd—N4	2.201 (10)	N3—C8	1.313 (14)
Cd—N2	2.238 (9)	N3—C6	1.392 (16)
Cd—I2	2.7248 (13)	N3—C5	1.544 (14)
Cd—I1	2.7358 (13)	C3—H3A	0.9300
C1—N1	1.429 (16)	N4—C8	1.315 (16)
C1—H1A	0.9600	N4—C7	1.369 (18)
C1—H1B	0.9600	C4—H4A	0.9300
C1—H1C	0.9600	C5—H5A	0.9600
N1—C4	1.355 (16)	C5—H5B	0.9600
N1—C2	1.377 (19)	C5—H5C	0.9600
N2—C4	1.279 (15)	C6—C7	1.360 (18)
N2—C3	1.372 (16)	C6—H6A	0.9300
C2—C3	1.33 (2)	C7—H7A	0.9300
C2—H2A	0.9300	C8—H8A	0.9300
			
N4—Cd—N2	112.2 (4)	C2—C3—N2	111.2 (14)
N4—Cd—I2	103.7 (3)	C2—C3—H3A	124.4
N2—Cd—I2	109.4 (3)	N2—C3—H3A	124.4
N4—Cd—I1	111.4 (3)	C8—N4—C7	104.1 (11)
N2—Cd—I1	101.1 (3)	C8—N4—Cd	122.5 (9)
I2—Cd—I1	119.20 (5)	C7—N4—Cd	133.3 (9)
N1—C1—H1A	109.5	N2—C4—N1	110.0 (12)
N1—C1—H1B	109.5	N2—C4—H4A	125.0
H1A—C1—H1B	109.5	N1—C4—H4A	125.0
N1—C1—H1C	109.5	N3—C5—H5A	109.5
H1A—C1—H1C	109.5	N3—C5—H5B	109.5
H1B—C1—H1C	109.5	H5A—C5—H5B	109.5
C4—N1—C2	108.3 (10)	N3—C5—H5C	109.5
C4—N1—C1	125.7 (12)	H5A—C5—H5C	109.5
C2—N1—C1	126.0 (12)	H5B—C5—H5C	109.5
C4—N2—C3	106.3 (12)	C7—C6—N3	106.8 (12)
C4—N2—Cd	124.4 (9)	C7—C6—H6A	126.6
C3—N2—Cd	129.2 (10)	N3—C6—H6A	126.6
C3—C2—N1	103.9 (13)	C6—C7—N4	109.3 (12)
C3—C2—H2A	128.0	C6—C7—H7A	125.3
N1—C2—H2A	128.0	N4—C7—H7A	125.3
C8—N3—C6	104.7 (10)	N3—C8—N4	114.9 (12)
C8—N3—C5	129.7 (10)	N3—C8—H8A	122.5
C6—N3—C5	125.6 (10)	N4—C8—H8A	122.5

### Hydrogen-bond geometry (Å, °)

**Table:**

*D*—H···*A*	*D*—H	H···*A*	*D*···*A*	*D*—H···*A*
C5—H5B···I1^i^	0.96	3.03	3.9797	169

Symmetry codes: (i) *x*−1/2, −*y*+3/2, −*z*+2.
